# A Local Stability Supported Parallel Distributed Constraint Optimization Algorithm

**DOI:** 10.1155/2014/734975

**Published:** 2014-07-03

**Authors:** Duan Peibo, Zhang Changsheng, Zhang Bin

**Affiliations:** School of Information Science & Engineering, Northeastern University, Shenyang 110819, China

## Abstract

This paper presents a new distributed constraint optimization algorithm called LSPA, which can be used to solve large scale distributed constraint optimization problem (DCOP). Different from the access of local information in the existing algorithms, a new criterion called local stability is defined and used to evaluate which is the next agent whose value needs to be changed. The propose of local stability opens a new research direction of refining initial solution by finding key agents which can seriously effect global solution once they modify assignments. In addition, the construction of initial solution could be received more quickly without repeated assignment and conflict. In order to execute parallel search, LSPA finds final solution by constantly computing local stability of compatible agents. Experimental evaluation shows that LSPA outperforms some of the state-of-the-art incomplete distributed constraint optimization algorithms, guaranteeing better solutions received within ideal time.

## 1. Introduction

Distributed constraint optimization problem (DCOP) is an effective framework used in solving multiagent system (MAS) problem, such as disaster rescue [1], sensor network [2], meeting scheduling [3], and peer-to-peer networks [4]. In order to solve DCOPs, several distributed optimization algorithms have been proposed. These algorithms can be divided into complete and incomplete algorithms. Since most of complete algorithms based on ADOPT [5] or DPOP [6] search the best solution with a lot of time, a series of incomplete algorithms have been developed. Some of them are based on the decisions of local groups of agents such as DALO [7], and KOPT [8]. Unfortunately, this kind of algorithms usually gets optimal solution over a long period of time because of guaranteeing the quality of the solution. On the other hand, some jobs have focused on making better decision by the information came from neighboring agents, such as DBA/DSA [9, 10].

Aiming at making better decision in the latter type of algorithms, one variable usually gets messages from its local variables. MULBS [11] is such outstanding algorithm. It uses most suitable features from both top-down and bottom-up algorithms, modifying the value of variable by judging whether the local solution can optimize the global solution. From the characteristics of these incomplete algorithms, the following basic questions we need to focus on: firstly, how to construct the initial solution? In the preprocessing of MULBS, the algorithm generated the whole partial candidate solution of variables in the agent. However, the preprocessing itself is an exponential problem; secondly, in order to refine the initial solution, we need a strategy to select next variable whose value should be changed instead of varying all variables since there are always such variables which step into a steady-state; and thirdly, when to terminate the algorithm?

To this end, this paper puts forward a new incomplete algorithm named LSPA which can obtain optimal or better suboptimal solution. In more details, this paper makes the following contributions.

- We show a better initial solution that can be treated as an optimal one of a DCOP with simple graph topology. We set up a pseudotree according to the degree of each node based on the constraint graph ensures that the degrees of leaves are the smallest. And then, we construct the initial solution from bottom to root by assigning values to agents without repetition.
- A new criterion named local stability is defined and used to evaluate which is the next agent whose value needs to be changed. We start to explore local search from the agents with low local stabilities. It is necessary to change their values if the quality of global optimal solution can be improved. After each refinement, we rearrange the local stabilities of agents based on their new assignments. In particular, the changes of the assignments are permitted to handle concurrently among compatible agents.
- We provide a termination detection mechanism built into the algorithm. The recalculation of local stability terminates whenever the whole agents step into a steady-state or the local stability of each agent is constant.

This paper is structured as follows. In Section 2 we give an overview of DCOP. Meanwhile, we analyze some incomplete DCOP algorithms based on individual agent. Next, in Section 3 we propose LSPA, a novel DCOP approximate algorithm through local stability of agent. In Section 4 we detail an empirical evaluation of the performance of LSPA against other algorithms by two groups of experiments. Finally, we draw some conclusions in Section 5.

## 2. Preliminaries

In this section, we formally define DCOP and introduce DSA/DBA and MULBS algorithms. Finally, we analyze the features of incomplete DCOP algorithms based on individual agent.

### 2.1. DCOP

A DCOP is usually modeled by 〈*A*, *X*, *α*, *D*, *F*〉, where *A* = {*a*_1_, *a*_2_,…, *a*_*v*_} is a set of agents; *X* = {*x*_1_, *x*_2_,…, *x*_*p*_} is a set of variables involved in agents; *α* : *X* → *A* denotes each *x*_*i*_ ∈ *X* just belongs to a unique agent *a*_*j*_ ∈ *A*; *D* = {*d*_1_, *d*_2_,…, *d*_*n*_} is a set of finite domains of *X*, such that *x*_*i*_ ∈ *X* takes values in *d*_*i*_ ∈ *D*; *F* = {*f*_1_, *f*_2_,…, *f*_*m*_} is a set of utility functions. A utility function *f*_*i*_ : *d*_*i*_1__ × *d*_*i*_2__ → *R* ^+^ ∪ *∞* represents the relevance for the variables of *a*_*i*_. The objective function of a DCOP is to calculate the cost of a complete assignment in which every variable that has assigned a value is the sum of all binary and unary cost functions evaluated on those values.

A practical issue will be set up as an undirected constraint graph by these five elements. The destination of solving DCOP is to find the best global assignment of variables instead of partial optimum solution. Most of DCOP algorithms firstly convert undirected constraint graph into a DFS pseudotree in the preprocessing. In the pseudotree, nodes correspond to variables and edges connect pairs of variables appearing in the same binary cost function of graph.

### 2.2. Incomplete DCOP Algorithms Based on Individual Agent

#### 2.2.1. DBA

In the DBA, neighboring agents exchange information by evaluating how much the solution could be improved if a local agent assignment was changed. Similar with other local search algorithms, the agents easily get stuck in local minima, in which no single local change seems to improve the solution. In order to solve this problem, researchers cast DBA into a DisWCSP [12] by increasing the weights of the current assignment. Based on this research direction, a lot of improved algorithms have been proposed later, such as IDB [13].

#### 2.2.2. DSA

The DSA is similar with the DBA which explores local search with a different strategy to escape from local minima. Probability is induced to justify whether there is an agent that can improve the quality of global solution through changing its value. In order to ensure the probability, a variant of the algorithms (A, B, C, D, or E) has been proposed. In [9], the method showed how both DSA and DBA could be generalized to DCOPs and compared their respective performances.

#### 2.2.3. MULBS

MULBS is an incomplete algorithm that optimally solves large scale DCOP, especially collaborative meeting scheduling. It mainly divided into the following two phases:

- generation of a global candidate solution
- refinement of the global candidate solution.

Before the execution of algorithm, the preprocessing will be done in order to generate pseudotree and find out the whole impossible partial candidate solutions of each agent. Partial candidate solutions of each agent are ordered from small to large based on the cost values. It is convenient for agent selecting optimal value in the search.

The generation of a global candidate solution starts with leaf agents choosing local values for their variables, and then they broadcast to parents. A higher priority agent receives PARTIAL_SOLUTION message that contains the assignments of children and chooses a local value from candidate solutions based on minimal conflict strategy. This partial solution is also broadcasted to its parent. The generation of the global candidate solution ends up when the root agent selects its value also minimizing conflicts.

In the refinement process, each agent searches its local space of partial solutions. The process starts with the root node through passing STORE_SOLUTION message from high to low priority nodes. If the merging between the global candidate solution and a partial solution generates a better global solution, it is propagated to the parent. If the root node receives a better solution, it updates the current solution and the global threshold and the process start again. The algorithm ends up when all the nodes in the pseudotree do not change their values.

### 2.3. Analysis of Incomplete Algorithms Based on Individual Agent

There are almost two phases in the incomplete algorithms whose assignment is based on the individual agent. In the first phase, an initial solution is usually constructed. And then the algorithm will refine the initial solution in the second phase. For instance, DSA or DBA assigns value to each agent at random in the first phase. Due to the serious stochastic, this initial solution could not be used as a suboptimal one. In order to improve the usability of the initial solution, MULBS algorithm assigns value to each agent based on the partial candidate solutions ordered by the costs with the strategy of minconflicts. For simple problems, such solution can be verified as the best global solution. However, every agent had to calculate and store all the partial candidate solutions ordered by their costs in the preprocessing. The calculation in this process will exponentially increase with the linear growth of the number of its own adjacent agents. For instance, the neighbors of *x*_1_ is {*x*_2_, *x*_3_,…, *x*_*n*_}. Assuming the domain of *x*_*i*_ is *D*_*i*_  (*i* ∈ 1,2,…, *n*), agent *x*_1_ stores num(*D*_1_) × num(*D*_2_) × ⋯×num(*D*_*n*_) possible partial candidate solutions which represent the Cartesian product *D*_1_ × *D*_2_ × ⋯×*D*_*n*_. In a dense constraint graph, it seems impossible to generate all the partial candidate solutions. As a result, the preprocessing of MULBS is only used in the constraint graph with low density. Note that the purpose of one incomplete algorithm is to solve large-scale DCOPs within an ideal time. The dense constraint graphs cannot be avoided.

**Figure 1:**
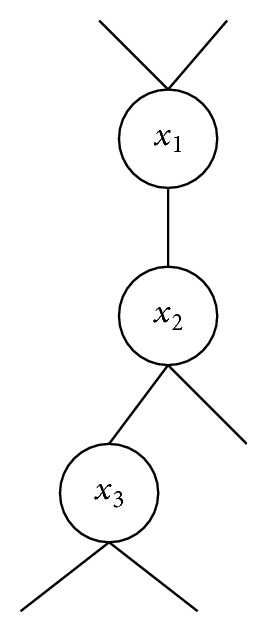
Portion of a pseudotree.

The termination of the distributed constraint optimization algorithms is also the key to determine the efficiency of algorithms. DBA/DSA adopts the strategy of distributed termination detection to finish local search. In order to avoid superfluous calculation, MUBLS finishes the second phase after traversing all the agents from root to leaves. Although it finds a better global solution in the local search, the best solution might be ignored. We give an example shown in Figure 1.

A portion of a pseudotree is presented in Figure 1. The priority from high to low of these three agents is 〈*x*_1_, *x*_2_, *x*_3_〉. When *x*_1_ makes local search, the current partial solution of *x*_1_ is {…, *x*_1_/*v*_1_, *x*_2_/*v*_2_,…}. For agent *x*_2_, a better partial solution has been generated by the assignment of {…, *x*_1_/*v*_1_, *x*_2_/*v*_2_,…}∪{*x*_3_/*v*_3_,…}. When the search came down to *x*_3_, the current partial solution was found with the assignment of {…, *x*_1_/*v*_1_,…}∪{…, *x*_2_/*v*_2_′, *x*_3_/*v*_3_′,…}. Assuming there is a potential best solution of {…, *x*_1_/*v*_1_′, *x*_2_/*v*_2_′, *x*_3_/*v*_3_′,…} when *x*_1_ = *v*_1_′, unfortunately, the algorithm cannot backtrack to *x*_1_. Therefore, the fact is that we neither traverse all the agents infinitely nor traverse them once like MULBS.

## 3. LSPA

The basic idea for obtaining optimal solution is to constantly modify the assignments of agents in order to close to the optimal one. In the process of altering the values of agents, more and more assignments of agents will tend to be stable. Even though we change the current value of one agent, it may be backtracked at some point in the future. We call such agent which difficultly gets an accurate assignment but seriously affects global optimal solution as key agent. Due to the limit information exchanged among agents, it is hard to ensure key agents. On the contrary, the existing of key agents results in expending a lot of time by changing assignments of some agents repeatedly in local search.

The key to solving this problem lies in the following aspects. First, a criterion is needed to gather enough information from neighboring agents in order to determine which agents tend to be stable. Second, we need to make sure which is the next agent whose assignment needs to be changed. At last, in order to improve the executive efficiency of algorithm, to some extent, we have to satisfy the parallelism of the algorithm. In this paper, we present an algorithm LSPA based on the local stability of agents to help selection and variation. Like DSA/DBA and MULBS, LSPA gets information by local search.

LSPA is mainly divided into two phases, the formation of initial solution based on a pseudotree and the refinement of global solution. The preprocessing has been accomplished before the implementation of the algorithm. Since there is a one-to-one relationship between an agent and its variable, for notation simplicity, we occasionally do not distinguish an agent and its variable. At the same time, we give the following definitions.

**Definition 1 (Partial Best Assignment (Vector)).**

Assuming the neighboring agents of one agent *x* in the constraint graph is *N* = {*n*_1_, *n*_2_,…,*n*_*k*_}, {*D*_1_, *D*_2_,…,*D*_*k*_} is the domain set corresponding to each agent in *N* · cost(*v*, *v*_*n*_*k*__) represents the cost calculated by the constraint between agent *x* and neighboring agent *n*_*k*_. One denotes Partial Best Assignment of *x* by *θ*(*x*) = {(*v*_1_, *v*_*n*_1__), (*v*_2_, *v*_*n*_2__),…, (*v*_*k*_, *v*_*n*_*k*__)}, Partial Best Assignment Vector by *π*(*x*) = 〈*v*_1_, *v*_2_,…, *v*_*k*_〉 which cost(*v*_*k*_, *v*_*n*_*k*__) = min⁡(cost(*v*, *v*_*n*_*k*__)).

**Definition 2 (Initial Partial Best Assignment).**

Assuming the neighboring agents of *x* is *N*, one says *θ*_*N*_(*x*) = {(*v*_1_, *v*_*n*_1__), (*v*_2_, *v*_*n*_2__),…, (*v*_*k*_, *v*_*n*_*k*__)} is Initial Partial Best Assignment of *x* when the following condition holds: ∀*v* ∈ *D*_*x*_, ∀*v*_*n*_*k*__ ∈ *D*_*n*_*k*__, cost(*v*_*k*_, *v*_*n*_*k*__) = min⁡(cost(*v*, *v*_*n*_*k*__)).

**Definition 3 (Current Partial Best Assignment).**

Assuming the neighboring agents of *x* is *N*, the assignments of *N* are *V* = {*v*_*n*_1__, *v*_*n*_2__,…, *v*_*n*_*k*__}; one says *π*_*N*_(*x*) = {*v*_1_, *v*_2_,…, *v*_*k*_} is Current Partial Best Assignment of *x* if cost(*v*_*k*_, *v*_*n*_*k*__) = min⁡(cost(*v*, *v*_*n*_*k*__)).

Next, one introduces the definition of local stability which is used as a criterion for receiving key agent.

**Definition 4 (local stability).**

The neighboring agents of *x* is *N*, Current Partial Best Assignment of *x* is *π*_*N*_(*x*). Assuming current value of *x* is *x*_current_, *τ*(*x*_current_) represents the number of neighboring agents who expect the assignment of *x* is *x*_current_. One denotes local stability of *x* by *s*(*x*_current_) if the following condition holds:

(1) $$\begin{array}{c} s\left(x_{\text{current}}\right)=\{\begin{array}{cc} 0, & \text{if}\;\;x_{\text{current}}\notin \pi_{V}\left(N\right)\\ \frac{\tau \left(x_{\text{current}}\right)}{\tau \left(N\right)}, & \text{if}\;\;x_{\text{current}}\in \pi_{V}\left(N\right). \end{array} \end{array}$$

**Definition 5 (compatible agent).**

For a given DCOP with constraint graph *G* = (*V*, *E*), *x*_1_ ∈ *V* and *x*_2_ ∈ *V*(*x*_1_ ≠ *x*_2_), *N*_1_ is neighboring agents of *x*_1_ and *N*_2_ is neighboring agents of *x*_2_, one says *x*_1_ and *x*_2_ compatible if *N*_1_∩*N*_2_ = *∅*.

In the preprocessing, we calculated *θ*(*x*) and *π*(*x*) of each agent. The costs calculated by the constraints between *x* and its neighbors are ordered and stored in the solution spaces. For generating a pseudotree, we do not randomly select a node as root. Instead we firstly traverse the constraint graph and get the degree of each node. Second, the node with highest degree is selected as the root. We will guarantee that the degrees of children nodes are smaller than their father's. It is easy to select appropriate values of leaves because the number of their neighbors is small.

**Figure 2:**
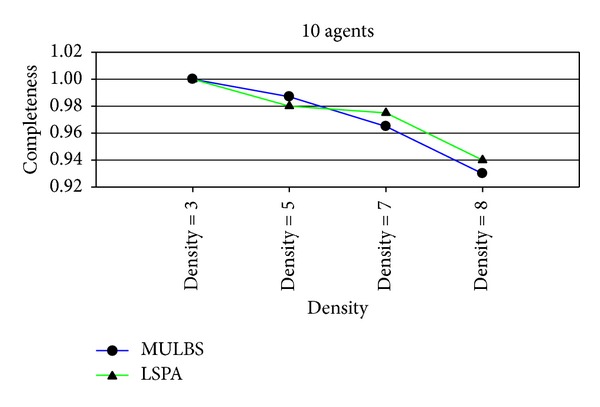
Completeness of initial solutions received by MUBLS and LSPA.

**Figure 3:**
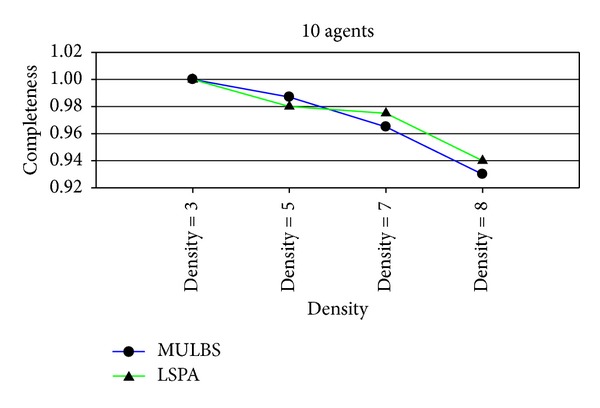
Run time of MUBLS and LSPA in the first phase.

In the first phase of the algorithm, we start to generate the initial solution from leaf agents. Two sets are used to store agents in different situations. The first one *S*_valued_ stores agents whose values have been assigned. The other one *S*_unvalued_ stores remaining nodes. At the beginning, *S*_unvalued_ stores all the nodes in the pseudotree. We randomly select one leaf agent in *S*_unvalued_ by assigning a value with the highest initial local stability in its domain. And then, the agent whose value has been assigned is put into *S*_valued_ from *S*_unvalued_ and the partial solution is broadcasted to the next agent in S_unvalued_. The construction of the initial solution terminates if *S*_unvalued_ = *∅*. We can say that such solution is an approximation optimal one of DCOP. From the experiments presented in Figures 2 and 3, we can observe that the quality of initial solutions is about the same with the ones received by MULBS. The experiments also show us that less run time is used by LSPA.

In the second phase of the algorithm, we refine the initial solution. LSPA will calculate local stability of each agent firstly. We confirm that the local solution of an agent will tend to be stable if the local stability of this agent is high since the local stability reflects the amount of its neighbors which expect it hold current value. Under this premise, we will change the value of the most unstable (with minimum local stability) agent if the quality of global solution can be improved. Otherwise, the old assignment will be retained. The new global solution is broadcasted to all the agents as a threshold. After that, we recalculate local stabilities and refine current global solution until the assignments of all agents are stable or local stabilities cannot be improved anymore. Because the values of the neighboring agents cannot be changed in each refinement, the compatible agents can explore local search asynchronously.

**Figure 4:**
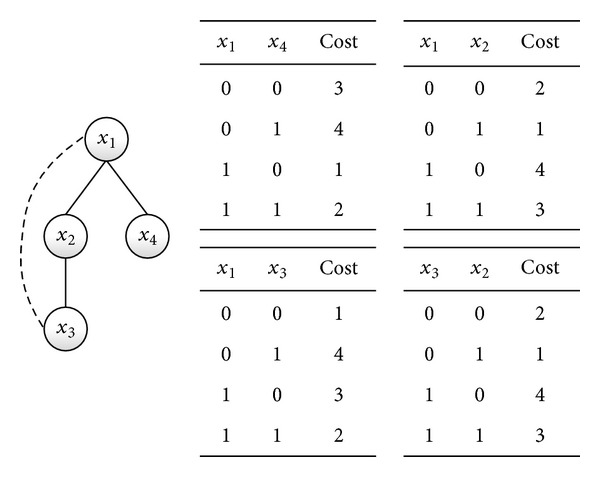
Constraints and pseudotree generated by degree.

**Figure 5:**
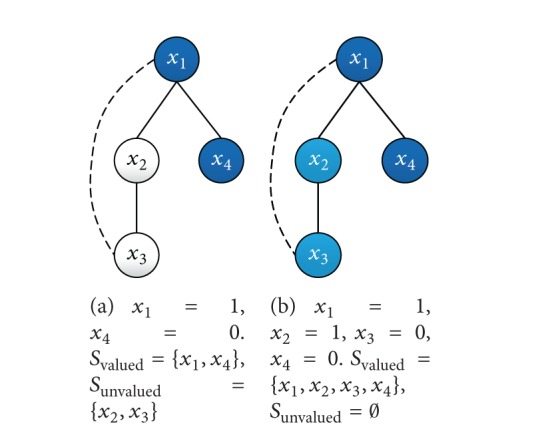
Construction of initial solution.

We give an example to illustrate the algorithm by a constraint graph with four agents. For convenience, we assume each domain of {*x*_1_, *x*_2_, *x*_3_, *x*_4_} is {0,1}. In the preprocessing (Algorithm 1 lines 1 to 14), LSPA firstly generated a pseudotree presented in Figure 4. And all the necessary variables were initialized and stored. As shown in the Figure 5(a), the initial solution has been constructed from leaf agent *x*_4_ with {*x*_4_ = 1, *x*_1_ = 0} because the initial local stability of *x*_4_ = 1 is 1 (Algorithm 1 lines 35 to 54). After that, the remaining agents *x*_3_ and *x*_2_ will be assigned values by the same method. The final initial global solution is {*x*_1_ = 1, *x*_2_ = 1, *x*_3_ = 0, *x*_4_ = 0} (Figure 5(b)).

In the refinement, each agent calculates its own local stability (Algorithm 1 lines 60 to 79). For instance, the neighbors of *x*_1_ is *N* = {*x*_2_, *x*_3_, *x*_4_}, the current stability of *x*_1_ is *s*(*x*_1_ = 1) = 1/3 calculated by *θ*_*N*_(*x*_1_) = {(0,1), (0,0), (1,0)}, and *π*_*N*_(*x*_1_) = 〈0,0, 1〉. Current local stabilities of other agents are *s*(*x*_2_ = 1) = 1, *s*(*x*_3_ = 0) = 1/2, and *s*(*x*_4_ = 0) = 1; the cost of current global solution is 8. A terminal detection executes before the local search if all the agents have reached the highest degree of stability. Otherwise *x*_1_ is selected to be the first node to change the value because its local stability is the lowest. When we change the value of *x*_1_ from 1 to 0, we find that its local stability will change to *s*(*x*_1_ = 0) = 2/3 and the cost of new global solution is 6 which is less than the current one. So we change the current value of *x*_1_. After that, the algorithm will terminate because all the agents have stepped into stable state with recalculated stabilities *s*(*x*_1_ = 0) = 2/3, *s*(*x*_2_ = 1) = 1, *s*(*x*_3_ = 0) = 1, and *s*(*x*_4_ = 0) = 1. The final global solution is {*x*_1_ = 0, *x*_2_ = 1, *x*_3_ = 0, *x*_4_ = 0}.

**Figure 6:**
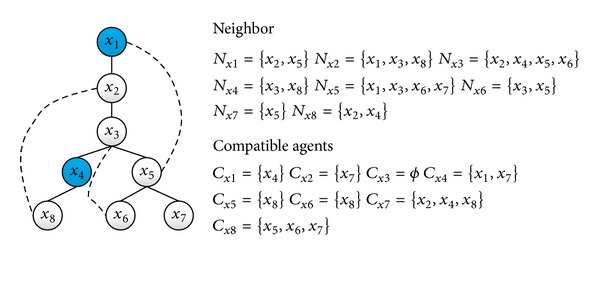
Parallelism based on a complex constraint graph.

A more complex constraint graph is given in Figure 6 in order to illustrate the parallelism of LSPA. According to, Figure 6 shows us neighboring and compatible agents set of each node. We assume that agent *x*_1_ has minimal local stability. The parallel local search will be implemented in *x*_1_ and *x*_4_ (Two nodes in deep color in Figure 6) because there are no public agents in their neighboring agents set.

## 4. Experiments

**Figure 7:**
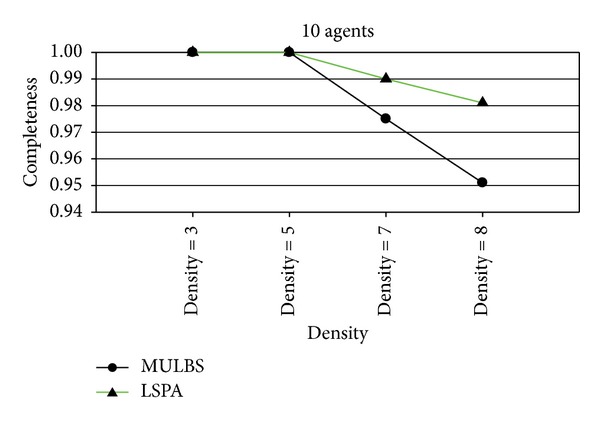
Completeness with 10 agents and densities 3, 5, 7, and 8.

**Figure 8:**
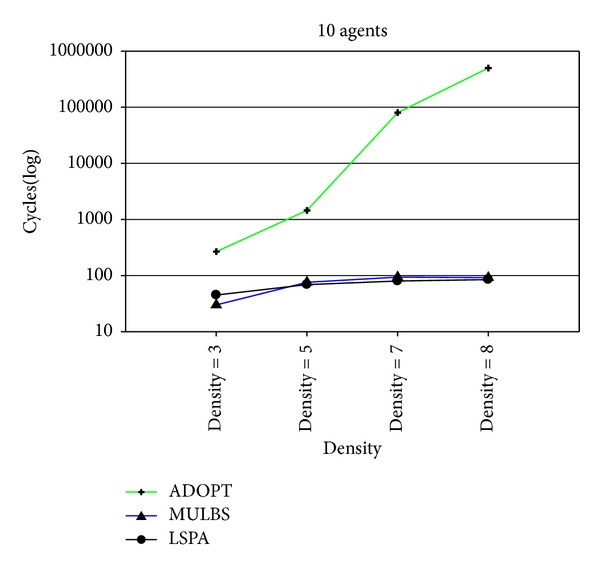
Cycles with 10 agents and densities 3, 5, 7, and 8.

**Figure 9:**
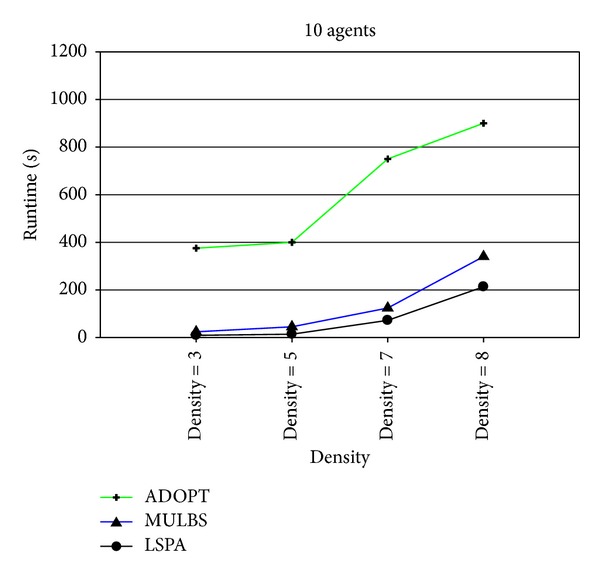
Runtime with 10 agents and densities 3, 5, 7, and 8.

**Figure 10:**
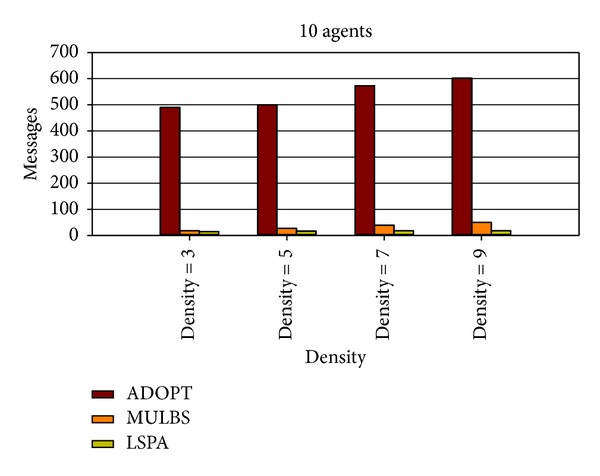
Messages with 10 agents and densities 3, 5, 7, and 8.

**Figure 11:**
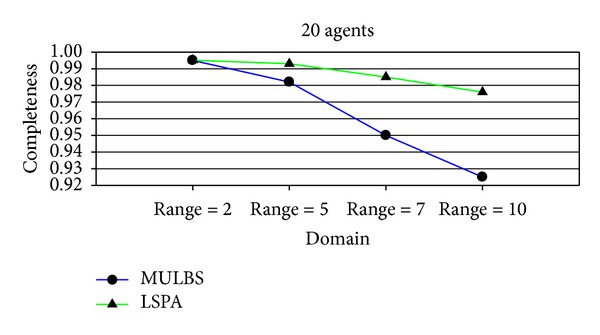
Completeness with density 3 and domains 2, 5, 7, and 10.

**Figure 12:**
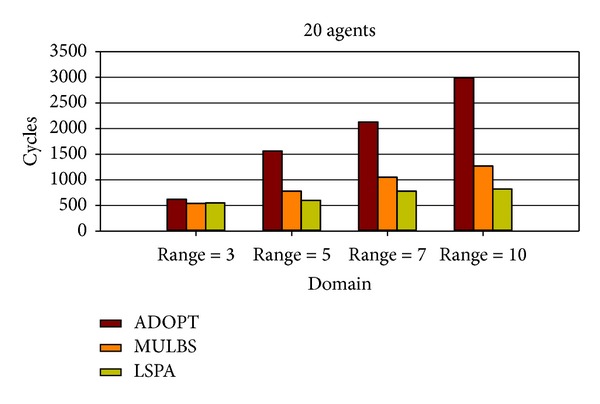
Cycles with density 3 and domains 2, 5, 7, and 10.

**Figure 13:**
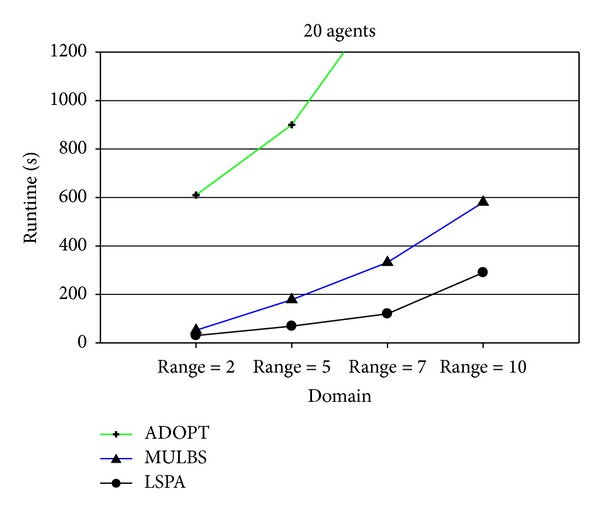
Runtime with density 3 and domains 2, 5, 7, and 10.

**Figure 14:**
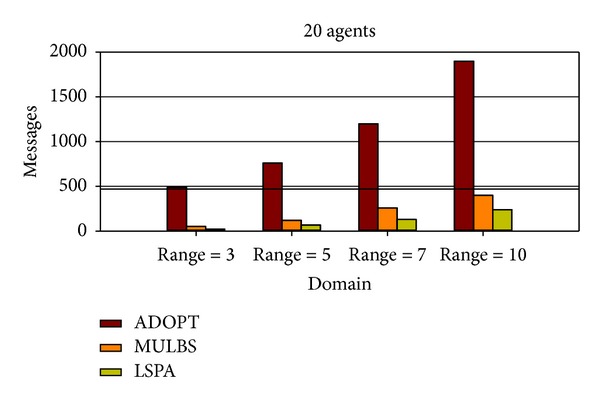
Messages with density 3 and domains 2, 5, 7, and 10.

At present, the evaluation metrics used in the experiment mainly refer to the run time and the number of messages. In particular, the run time of MULBS and LSPA includes preprocessing time. We adopt the metric of the completeness and the cycles as well in order to measure the quality of solutions and the concrete implementation of the algorithms. We see such a solution solved by ADOPT as a best cost. So the completeness is only compared between MULBS and LSPA. The performance of an incomplete algorithm is under the influence of two main features. One is the density related with the number of constraints and nodes in a constraint graph. The other is the domain size of each agent. The experiments in this paper are divided into two groups. The first group is presented in Figures 7–10; we start evaluating the behavior of the algorithms in a scenario with 10 agents and different densities (3, 5, 7, and 8). The second group is presented in Figures 11–14; we start evaluating the behavior of the algorithms in a scenario with 20 agents and different domains (2, 5, 7, and 10) with density 3.

Figures 7 and 11 are the measurement for the completeness of the solutions received by MULBS and LSPA. We observed that the completeness of both of the two algorithms decreased when graph density and variable domain size became larger since the structure of graph topology is complicated. However the drop rate of the solution quality got by LSPA is less than MULBS. We can get the conclusion that LSPA will get better solution compared with MULBS.

Figures 8 and 12 provide the cycles of ADOPT, MULBS, and LSPA algorithms. In this paper, a cycle is defined as all the agents have sent messages to neighbors and received messages from other agents. Since the message processing mechanisms are different in these algorithms, the time needed in one cycle is different. In order to be more effective to compare DCOP algorithms, the cycles will be used as an important criterion. As the analysis above, the agents in MULBS and LSPA algorithms transmit and process messages based on the adjacent variables. It can be used to explain the phenomenon that the cycles of MULBS and LSPA are almost the same in Figures 8 and 12. However the experimental result shows that the complete DCOP algorithms like ADOPT will use more cycles because ADOPT has to exchange more messages to get final solutions with quality guaranteed.

The results presented in Figures 9 and 13 show that the run time of LSPA is less than other two algorithms. In particular, the change of MULBS has been great in Figure 13 because of the exponential growth of partial candidate solutions in the preprocessing.

As what we have analyzed in former sections, both LSPA and MULBS get messages from neighbors. So a linear relationship exists between the total messages and the edges of the pseudotree in Figures 10 and 14. However, the messages of LSPA are less than MULBS because we avoid changing values repeatedly caused by key agents. Different from LSPA and MULBS algorithms, agents in ADOPT need more messages for modifying current solution.

## 5. Conclusions

In this paper we developed a new algorithm called LSPA. The same with some other incomplete algorithms, LSPA explores local search between the agent and its neighboring ones. A new criterion named local stability is used in the algorithm in order to justify which is the next agent whose assignment needs to be changed. The local stability represents the expectation of how neighboring agents want the local agent keep or change the current value. If the local stability of an agent with current assignment is high, we had better not change its value because it has stepped into a stable state. On the opposite, the agents with low local stability mean such agents may be key agents in the graph. Furthermore, the local search can execute concurrently among compatible agents because we do not change the value of its neighboring agents. The results we obtained from experiments are encouraging. Compared with ADOPT and MULBS, the advantages of LSPA are obvious in all kinds of metrics.

## Figures and Tables

**Algorithm 1:**
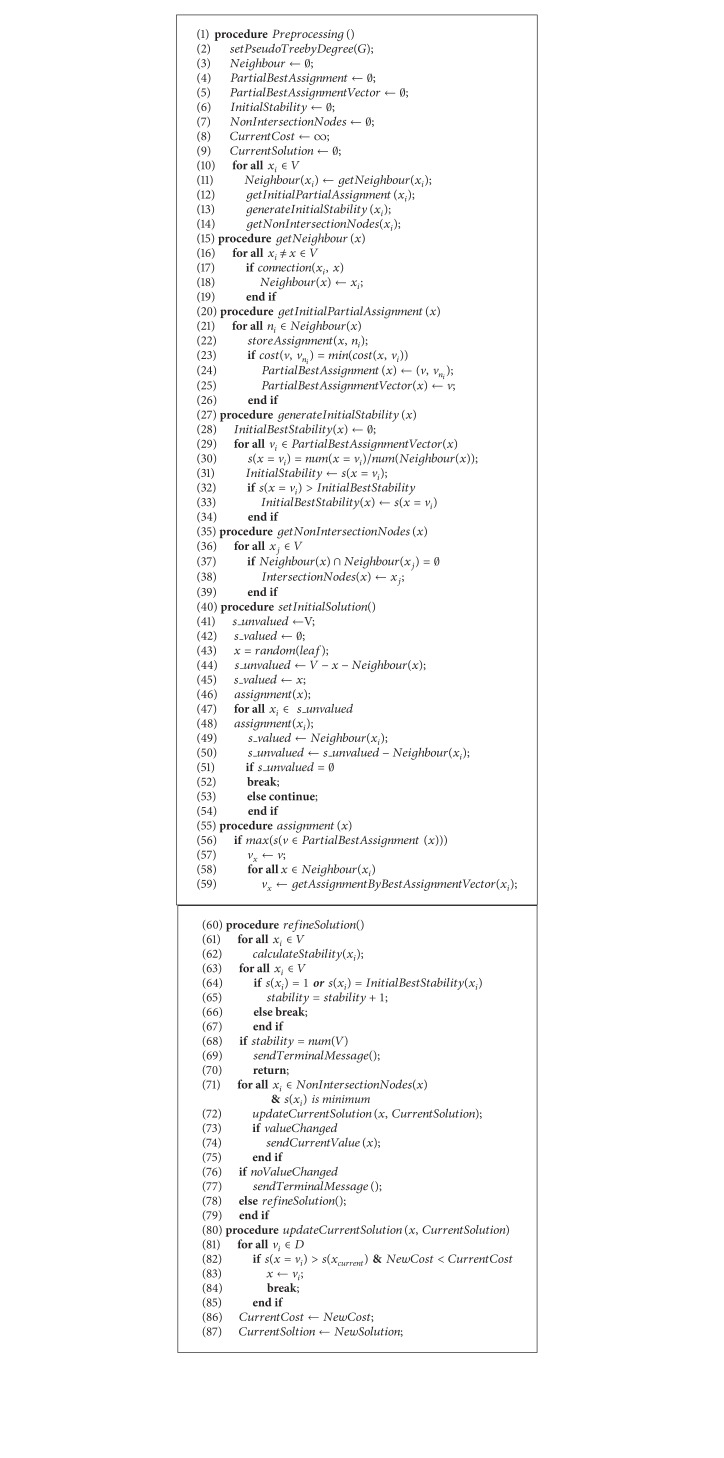
LSPA-main functions.
